# Diagnostic and Therapeutic Challenge of Metatarsalgia in a Patient With Rheumatoid Arthritis

**DOI:** 10.7759/cureus.21751

**Published:** 2022-01-30

**Authors:** João N Malta, Joana Martins, António Azenha, Pedro Lemos Pereira

**Affiliations:** 1 Medicina Física e de Reabilitação, Centro Hospitalar e Universitário de Coimbra, Coimbra, PRT

**Keywords:** synovitis, rehabilitation, morton neuroma, rheumatoid arthritis, metatarsalgia

## Abstract

A 63-year-old female patient, with a past history of rheumatoid arthritis, presented with insidious pain on the left foot second and third metatarsophalangeal joints, associated with swelling and morning stiffness (mean time: four hours). Physical examination evidenced a tender and soft nodularity in the third intermetatarsal space, along with sharp pain, consistent with Morton’s neuroma. Foot ultrasound suggested Morton’s neuroma, but not excluding the possibility of rheumatoid arthritis involvement. Foot magnetic resonance imaging suggested the possibility of extensive synovitis of the third metatarsophalangeal joint, but not excluding the coexistence of Morton’s neuroma because of the mass effect. Finally, the patient underwent an ultrasound-guided needle biopsy of the nodule, which confirmed metatarsophalangeal joint synovitis. The foot is a common location of rheumatoid arthritis manifestation, and metatarsophalangeal joint synovitis can mimic Morton’s neuroma. After a definite diagnosis, the patient recovered lower limb functional impairment after introducing adalimumab and a rehabilitation program. This case highlights the importance of an accurate differential diagnosis, pharmacological rheumatoid arthritis control, and physical medicine and rehabilitation programs to optimal clinical and functional improvement.

## Introduction

Metatarsalgia refers to pain generally located under one or more metatarsal heads [1]. Forefoot pain includes pain located in the toes and metatarsophalangeal joints [2]. Those locations are among the most common foot pain locations [1,2]. Manifestations of forefoot pain may be similar and a thorough assessment of pain location and associated clinical manifestations is key to differential diagnosis [2]. The causes include plantar callosities, plantar warts, subluxation, dislocation or synovitis of the metatarsophalangeal joints, hallux valgus, hallux rigidus, Freiberg disease, interdigital (Morton’s) neuroma, systemic disorders (such as rheumatoid arthritis, psoriatic arthritis, or gout), structural malalignment of the foot, sesamoiditis, stress fractures and trauma [1,2]. The foot is the second most common location of rheumatoid arthritis manifestation [3]. Foot pain, especially forefoot pain, and rheumatoid forefoot deformity are debilitating and a common reason why these patients search for medical care [3]. Synovitis leads to capsular distension and initiates bone and soft tissue destruction [3-5]. Loss of articular integrity and associated muscle imbalances result in dorsal subluxation of the proximal phalanx, hallux valgus, claw toe deformity, and plantar callosities, and associated with other conditions such as Morton’s neuroma [3-5]. The treatment mainstay is pharmacological rheumatoid arthritis control; notwithstanding it may be necessary other nonoperative measures such as the use of orthoses, or even surgical management [3,4]. The authors report a case of a rheumatoid arthritis patient who presented with metatarsalgia that revealed a challenging differential diagnosis between metatarsophalangeal joint synovitis and Morton’s neuroma, and its implications to therapeutic decision-making.

## Case presentation

A 63-year-old Portuguese female patient, with a past history of rheumatoid arthritis diagnosed in 2005, osteoporosis, and hypercholesterolemia, medicated with methotrexate, folate, alendronic acid, cholecalciferol, and atorvastatin. The patient presented with insidious pain evolving through 3 months on the second and third metatarsophalangeal joints of her left foot, associated with swelling and about 4-hour morning stiffness. Physical examination evidenced painless second and third metatarsophalangeal joint deformity and a tender and soft nodularity in the third intermetatarsal space, with sharp pain consistent with Morton’s neuroma. There was no history of trauma or another joint deformity, instability, or diminished range of movement. There were no inflammatory signs or plantar callosities within the skin, nor vascular insufficiency or neurologic deficits within the lower limbs. Disease activity according to disease activity score 28 (DAS28) was low.

**Figure 1:**
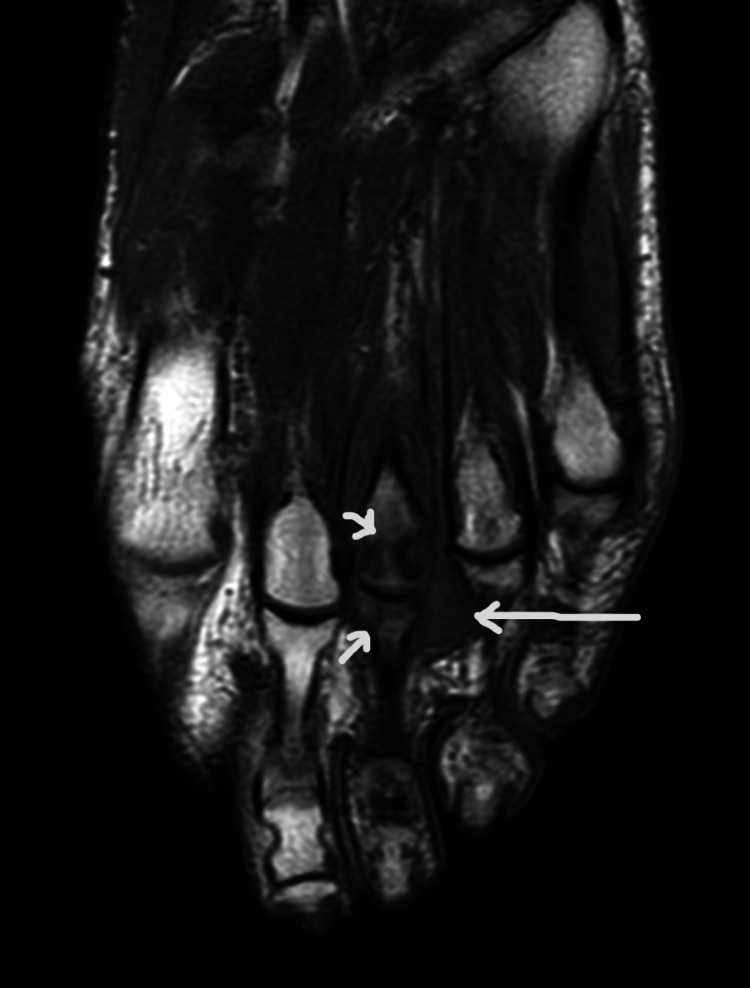
T1-weighted long-axis left-foot magnetic resonance imaging showing marked bone edema of the third metatarsal bone and the third proximal phalanx, and iso-signal widening of the third intermetatarsal space.

**Figure 2:**
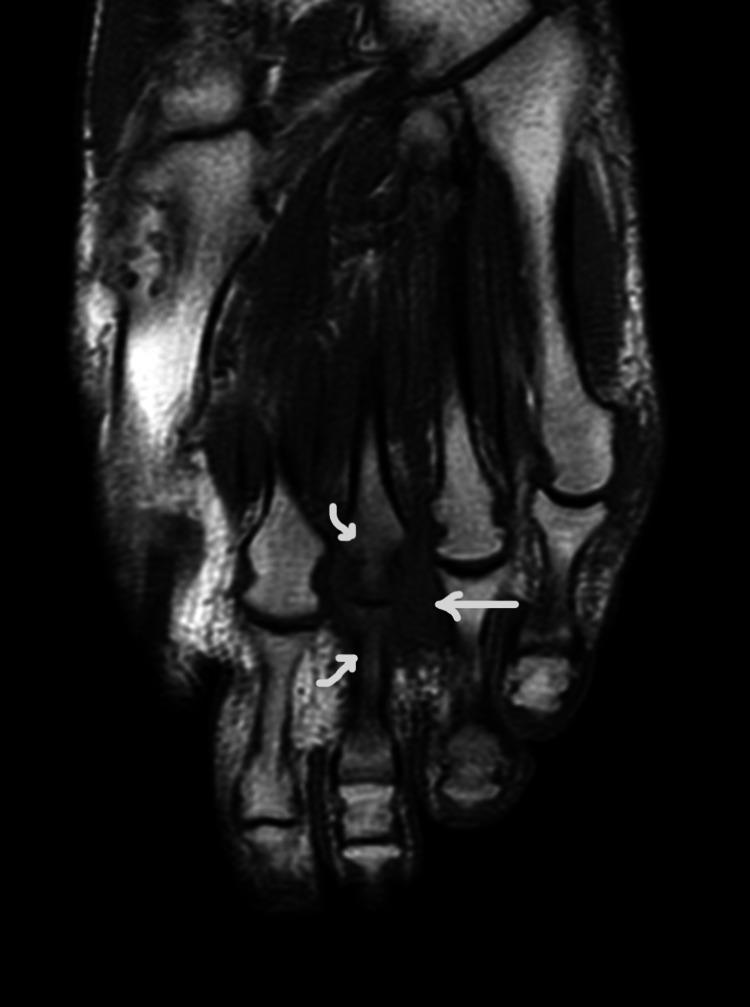
T2-weighted long-axis left-foot magnetic resonance imaging showing marked bone edema of the third metatarsal bone and the third proximal phalanx, and heterogeneous signal widening of the third intermetatarsal space.

There was no seric inflammatory markers elevation. Initially, the patient underwent a foot ultrasound that showed a hypoechogenic and heterogeneous nodule with 23x16x9 mm with vascularity, suggesting Morton’s neuroma, not excluding the possibility of rheumatoid arthritis involvement. At this phase, it had been initiated pharmacologic therapy with sulfasalazine, to counter rheumatoid arthritis involvement, with poor response and progressively worse lower limb functional impairment. Subsequently, the patient underwent left foot magnetic resonance imaging that showed marked bone edema of the distal third metatarsal bone and the third proximal phalanx, and a 18x10x17 mm widening of the third intermetatarsal space with T1-weighted iso-signal and T2-weighted heterogeneous signal, seemingly connected to the dorsal aspect of the third metatarsophalangeal joint, suggesting the possibility of extensive synovitis of this joint, but not excluding the coexistence of Morton’s neuroma because of mass effect (Figures 1-2). The doubt remained, so the patient underwent an ultrasound-guided needle biopsy of the nodule, which revealed fibrous connective tissue with mild inflammatory infiltrate and fibrin, compatible with synovitis.

Treatment was initiated with sulfasalazine 500 mg bid. However, three months later the pain did not subside and was associated with functional lower limb impairment. So, sulfasalazine dose was increased and the patient initiated an outpatient rehabilitation program with cryotherapy, third metatarsophalangeal joint and space-pulsed ultrasound therapy, slow passive range of motion exercises, and deep transverse friction massage to counter joint stiffness and maintain its range of motion, intrinsic foot muscles isometric strengthening exercises and gait training with a foot orthosis with retrocapital support. Two months later she presented with significant but not complete pain relief. At that time, with a definite diagnosis of metatarsophalangeal synovitis with ultrasound-guided needle biopsy, it was decided to initiate biweekly adalimumab while she maintained the same rehabilitation program.

The patient was evaluated about one month after beginning biological therapy. She reported sustained pain relief and no side effects. There were no signs of metatarsophalangeal joint arthritis with physical examination and it was initiated gradual discontinuation of sulfasalazine. Three months later, she presented with no pain and no lower limb functional impairment, with a normal gait, and low disease activity. Then the rehabilitation program was terminated and the patient continued gradual discontinuation of sulfasalazine.

## Discussion

The main differential diagnosis for metatarsalgia in this patient was between metatarsophalangeal synovitis and Morton’s neuroma, two common causes of metatarsalgia in patients with rheumatoid arthritis. Metatarsalgia differential diagnosis is usually possible with a careful medical history and physical examination [2]. In this case, therapeutic options would be heavily influenced by the definite diagnosis, given the different approaches to these two main differential diagnoses [3,6]. Clinical history, especially the presence of morning stiffness and joint swelling, was suggestive of metatarsophalangeal joint synovitis. However, physical examination findings, absence of blood inflammatory markers elevation, and poor response to sulfasalazine were more in favor of Morton’s neuroma hypothesis. Magnetic resonance imaging was suggestive of extensive synovitis of the third metatarsophalangeal joint, however not excluding coexistent Morton’s neuroma. The final diagnosis was distinguished recurring to the ultrasound-guided needle biopsy of the nodule, which confirmed metatarsophalangeal joint synovitis.

Metatarsophalangeal joint synovitis is often the initial manifestation of rheumatoid arthritis, and approximately two-thirds of rheumatoid arthritis patients have metatarsophalangeal joint involvement within the first three years [4]. This joint synovitis is accepted as the initiating agent of bone and soft tissue destruction that results in joint instability and associated muscle imbalances, which will eventually lead to rheumatoid forefoot deformity [3-5]. However, the feet may be overlooked in rheumatoid arthritis patients [3]. Of the American College of Rheumatology recommended disease activity measures [7], neither includes the feet in joint counts. Nonetheless, a physical metatarsophalangeal joint examination may be important, since a cross-sectional study showed a 79% correlation between physical examination and magnetic resonance imaging, mostly when the physical examination is normal [8]. In this presented case, DAS28 indicated low disease activity and the patient had pain and important lower limb functional impairment due to rheumatoid arthritis involvement.

According to Chaganti et al [9], rheumatoid synovitis and nodules mimicking Morton’s neuroma are rare. They describe five feet with rheumatoid nodules with a clinical presentation resembling Morton’s neuroma. Four of them had coexistent Morton’s neuroma, and the remaining foot had no neuroma at all. Neuromas had surgical treatment and the patients were referred to proper rheumatoid arthritis control.

Zielaskowski et al [10] described a case of multiple coexistent neuromas with synovitis and one rheumatoid nodule. The patient presented with pain and burning sensation at the third intermetatarsal space and morning stiffness within feet and hands from 30 minutes to one hour. She was initially treated with neuroma infiltration, with no relief, so she underwent surgery. Two masses were excised, one from the second intermetatarsal space, and another from the third intermetatarsal space. Both were consistent with coexistent Morton’s neuroma and chronic rheumatoid synovitis, and the mass from the third intermetatarsal space had findings consistent with rheumatoid nodules. Surgical excision resulted in complete pain relief.

Metatarsophalangeal joint synovitis and Morton’s neuroma can overlap and it can be confusing to separate them [11]. When the metatarsophalangeal joint is inflamed, the soft tissues around can be affected, including the interdigital nerves [11]. Furthermore, the neuroma can irritate adjacent structures [11]. The clinical history suggests the diagnosis [4,11]. Pain location, pain quality, chronology, and aggravating and alleviating factors may be the key to the diagnosis [4]. Morton’s neuroma is suggested by pain not located to the metatarsal head, aggravated with tight shoes or high heels, whereas metatarsophalangeal joint synovitis is suggested by pain on weight-bearing, associated with joint swelling, that alleviates with antiinflammatory medication [6,11]. Physical examination should focus on feet inspection, looking for deformities, the foot arches, callosities and swelling, and feet palpation, particularly the intermetatarsal spaces [4]. Other specific tests may be helpful, such as Mulder’s test and the drawer test [4]. Palpable clicking with Mulder’s test is extremely sensitive to Morton’s neuroma diagnosis [6]. A drawer test that reproduces the patient’s pain suggests metatarsophalangeal synovitis [11]. Miller [11] also suggested a differential injection with anesthetic into the intermetatarsal space, proximal to the metatarsal head, and into the metatarsophalangeal joint, alternately, and evaluate and compare the clinical response to distinguish between Morton’s neuroma and metatarsophalangeal joint synovitis.

Diagnosis of Morton’s neuroma is made clinically [4,6]. If doubt remains after a thorough clinical history and physical examination, imaging may be extremely helpful. Plain radiographs can be useful to assess deformities or fractures, ruling out other causes of pain [4,6,11]. Ultrasound scan and magnetic resonance imaging can be useful if Morton’s neuroma diagnosis is doubtful [4].

As mentioned before, rheumatoid forefoot treatment's mainstay is pharmacological rheumatoid arthritis control. This patient was previously medicated with methotrexate. According to the 2021 American College of Rheumatology Guideline for the Treatment of Rheumatoid Arthritis, there is a conditional recommendation to add a biologic or targeted synthetic disease-modifying antirheumatic drug over adding sulfasalazine and hydroxychloroquine [12]. In this patient, it was initiated sulfasalazine when the diagnosis was not clear, and adalimumab was introduced after that. Other conservative measures include the use of foot orthoses, which can help with forefoot pain control, but the available evidence shows conflicting results [3]. Surgical management of rheumatoid forefoot may be necessary to restore ambulation [3]. There are numerous possible procedures, with variable rates of success [3]. One classical procedure is a resection arthroplasty of the metatarsophalangeal joints [3]. Other procedures include first metatarsophalangeal fusion, in isolation or combined with the aforementioned arthroplasty [3]. Other techniques include joint-preserving surgery, consisting of metatarsal osteotomy and soft tissue reconstruction, with metatarsophalangeal joint function preservation [3,13]. Debridement of plantar callosities may offer some pain relief [3]. Synovectomy may have a role but is not usually performed because most patients present already with the consequent forefoot deformity [3].

On the other hand, Morton’s neuroma conservative treatment consists of shoe modification and a plantar orthosis with metatarsal unloading [2,6]. In about 30% of cases, conservative treatment and steroid injection provide long-term pain relief [6]. The remaining patients should be considered for surgical excision of Morton’s neuroma [6].

## Conclusions

The foot is a common location of rheumatoid arthritis manifestation and should not be overlooked. Metatarsophalangeal joint synovitis can mimic Morton’s neuroma, and Morton’s neuroma can coexist with metatarsophalangeal joint synovitis. Given the different approaches to the different clinical entities, an accurate diagnosis is mandatory to guide treatment. Clinical history and physical examination are important to the differential diagnosis of metatarsalgia and forefoot pain and, in doubtful cases, the diagnostic investigation must be exhaustive, as it was in this case, until a clear diagnosis is reached. After an accurate diagnosis, pharmacological rheumatoid arthritis control and physical medicine and rehabilitation programs are crucial to optimal clinical and functional improvement.
